# A large nested association mapping population for breeding and quantitative trait locus mapping in Ethiopian durum wheat

**DOI:** 10.1111/pbi.13062

**Published:** 2019-02-09

**Authors:** Yosef G. Kidane, Cherinet A. Gesesse, Bogale N. Hailemariam, Ermias A. Desta, Dejene K. Mengistu, Carlo Fadda, Mario Enrico Pè, Matteo Dell'Acqua

**Affiliations:** ^1^ Institute of Life Sciences Scuola Superiore Sant'Anna Pisa Italy; ^2^ Bioversity International Addis Ababa Ethiopia; ^3^ Amhara Regional Agricultural Research Institute (ARARI) Adet Agricultural Research Center Bahir Dar Ethiopia; ^4^ Department of Dryland Crop and Horticultural Sciences Mekelle University Mekelle Ethiopia

**Keywords:** quantitative trait loci, tetraploid wheat, multiparental mapping, GWAS, breeding, smallholder farmers

## Abstract

The Ethiopian plateau hosts thousands of durum wheat (*Triticum turgidum* subsp. *durum*) farmer varieties (FV) with high adaptability and breeding potential. To harness their unique allelic diversity, we produced a large nested association mapping (NAM) population intercrossing fifty Ethiopian FVs with an international *elite* durum wheat variety (Asassa). The Ethiopian NAM population (EtNAM) is composed of fifty interconnected bi‐parental families, totalling 6280 recombinant inbred lines (RILs) that represent both a powerful quantitative trait loci (QTL) mapping tool, and a large pre‐breeding panel. Here, we discuss the molecular and phenotypic diversity of the EtNAM founder lines, then we use an array featuring 13 000 single nucleotide polymorphisms (SNPs) to characterize a subset of 1200 EtNAM RILs from 12 families. Finally, we test the usefulness of the population by mapping phenology traits and plant height using a genome wide association (GWA) approach. EtNAM RILs showed high allelic variation and a genetic makeup combining genetic diversity from Ethiopian FVs with the international durum wheat allele pool. EtNAM SNP data were projected on the fully sequenced AB genome of wild emmer wheat, and were used to estimate pairwise linkage disequilibrium (LD) measures that reported an LD decay distance of 7.4 Mb on average, and balanced founder contributions across EtNAM families. GWA analyses identified 11 genomic loci individually affecting up to 3 days in flowering time and more than 1.6 cm in height. We argue that the EtNAM is a powerful tool to support the production of new durum wheat varieties targeting local and global agriculture.

## Introduction

Durum wheat (*Triticum turgidum* subsp. *durum*, genome AB, 2*n* = 4× = 28) is cultivated worldwide for traditional and high‐value food preparations thanks to its capacity to produce hard grains with remarkable nutritional value. World durum wheat production in 2016–2017 was an estimated 39.9 million tonnes, which is approximately 5% of global wheat production (International Grains Commission data). Nevertheless, durum wheat is a cultural crop of major importance in the Mediterranean basin, Central Asia, Central America, and Sub‐Saharan Africa. For this reason, durum wheat breeding efforts have been aimed at both quality and productivity traits. In the 1910s, the work of Nazareno Strampelli incorporated landrace germplasm with early varieties producing wheat lines that are still grown today (notably, the variety “Cappelli”). In the second half of the 20th century, Norman Borlaug's work introduced dwarfing genes into wheat breeding germplasm, increasing grain yield in durum *elite* lines that are still widely employed (Ortiz *et al*., 2007). Semolina quality traits, including pigmentation and gluten structure, were also the object of molecular breeding in durum wheat and, as such, are undergoing continuous improvement (Fiedler *et al*., 2017; Reimer *et al*., 2008).

Durum wheat germplasm is highly diverse (Kabbaj *et al*., 2017). In the Ethiopian highlands, where it has been cultivated by smallholder farmers for thousands of years, durum wheat has developed several traits of phenotypic (Mengistu *et al*., 2018) and molecular (Mengistu *et al*., 2016) uniqueness. In Ethiopia, durum wheat landraces –referred to here as farmer varieties (FVs) – are cultivated on 70% of the total wheat area, which is estimated at 2 million hectares (Hammer and Teklu, 2008). Ethiopian durum wheat is mostly grown in marginal environments with little use of chemical inputs and irrigation, and is at the base of many traditional preparations and a staple crop in the highlands (Tsegaye and Berg, 2007). Thousands of FVs evolved in Ethiopia with the simultaneous contribution of natural and artificial selection, following a complex pattern of trait selection and amplification (Kidane *et al*., 2017b; Mancini *et al*., 2017). Therefore, the Ethiopian durum wheat is a source of several adaptive traits of breeding relevance, including disease resistance alleles to rusts and Septoria (Kidane *et al*., 2017a; Liu *et al*., 2017; Zhang *et al*., 2017).

Globally, durum wheat is a source of beneficial alleles for biotic (Aoun *et al*., 2017) and abiotic (Peleg *et al*., 2009b; Tuberosa and Maccaferri, 2015) stresses also relevant to the bread wheat (*Triticum aestivum*, genome ABD, 2*n* = 6*x* = 42) gene pool. With its intermediate tetraploid karyotype, durum wheat may be crossed with wild wheats to incorporate their haplotypes in a cultivated background (Avni *et al*., 2014; Peleg *et al*., 2009a; Zhu *et al*., 2016), supporting the availability of novel allelic diversity to wheat breeders. The AB genomes of durum wheat can be hybridized with wild species such as *Aegilops tauschii*, the donor of the D genome, to create synthetic hybrids targeting adaptive traits (Reynolds *et al*., 2007, 2015). Alleles coming from *Triticum urartu*, the wild donor of the A wheat genome that still grows in highly differentiated natural populations (Brunazzi *et al*., 2018), have also been successfully introgressed in durum wheat amphiploids and lines (Alvarez *et al*., 2009). The closely related wild emmer (*Triticum dicoccoides*, genome AB, 2*n* = 4*x* = 28) may also be used to accelerate the introduction of novel diversity into wheat breeding through durum wheat (Valkoun, 2001). Genetic and genomic tools developed in durum wheat are increasingly integrated with those of bread wheat (Maccaferri *et al*., 2015). The current genomic revolution in wheat, which has seen the development and release of the genome sequence of several *Triticum* species including bread wheat (The International Wheat Genome Sequencing Consortium, 2018), wild emmer wheat (Avni *et al*., 2017), and diploid wheats (Ling *et al*., 2018; Wang *et al*., 2017), provides new tools for the characterization of the genetic basis of complex traits and for the fruitful exploitation of untapped allele pools (Uauy, 2017).

Understanding the genetic basis of quantitative traits, quantitative trait loci (QTL), is essential to achieve predictive wheat improvement. However, limitations in allelic diversity and genetic mapping resolution of currently available durum wheat mapping populations pose a limit to the capacity to pinpoint genes responsible for traits of agronomic interest. While diversity panels employed in genome‐wide association (GWA) studies increase the diversity available to search for causative alleles of interest, their historical genetic recombination history is unknown and does not allow the elevated statistical power of classical pedigree‐based haplotype mapping (Korte and Farlow, 2013; Mackay *et al*., 2009). Multiparental population designs have been created to bridge the two approaches, incorporating higher genetic diversity and recombination frequencies in pedigree‐based crosses (Churchill *et al*., 2004; Ladejobi *et al*., 2016), and are developed following two main approaches. Multiparental advanced generation intercross (MAGIC) populations are produced by crossing four or more founder lines in a balanced scheme (Huang *et al*., 2015). MAGIC populations have been developed in several cereal species including maize (Dell'Acqua *et al*., 2015), wheat (Mackay *et al*., 2014; Milner *et al*., 2016), rice (Bandillo *et al*., 2013), and barley (Sannemann *et al*., 2015). Conversely, nested association mapping (NAM) populations are produced by intercrossing one recurrent founder line with *n* other founder lines, which results in *n* recombinant inbred line (RIL) families that share the recurrent founder haplotype. The NAM design was developed in maize (McMullen *et al*., 2009) and later applied to several maize genetic backgrounds (Bauer *et al*., 2013) and different crops including soybean (Li *et al*., 2017), sorghum (Bouchet *et al*., 2017), barley (Maurer *et al*., 2015; Nice *et al*., 2016), and bread wheat (Bajgain *et al*., 2016; Jordan *et al*., 2018).

The NAM design has the merit of mirroring breeding approaches aimed at incorporating traits of choice in a background of choice, following a star design. A NAM population can therefore play the double role of research tool and breeding tool. In the case of Ethiopian durum wheat, a large gap exists between the genetic makeup of local FVs and modern varieties (MVs) released for cultivation in the country as demonstrated by their genotypic (Kabbaj *et al*., 2017; Mengistu *et al*., 2016) and phenotypic diversity (Mengistu *et al*., 2018). The Ethiopian durum wheat allele pool is unique with respect to international breeding material, and its incorporation into breeding efforts could benefit both local and global farmers (Kidane *et al*., 2017b; Mancini *et al*., 2017).

In this work, we describe the development of a large Ethiopian durum wheat NAM (EtNAM) population built by intercrossing of a MV with international background with 49 Ethiopian FVs and one Italian MV, which were chosen on the basis of their phenotypic and molecular diversity. A subset of 12 EtNAM families, 100 RILs each, was genotyped with 13 000 single nucleotide polymorphisms (SNPs), allowing the characterization of the genetic diversity and structure of the population. We conclude by conducting a GWA study for phenology and plant height, reporting a number of loci accounting for a relevant amount of the phenotypic variation in the EtNAM. We make the population available to the wheat community, and argue that the EtNAM may act as a breeding tool and a research tool that supports the identification of QTL of agronomic relevance and their incorporation into pre‐breeding materials.

## Results

### Production and diversity of the EtNAM population

**Figure 1:**
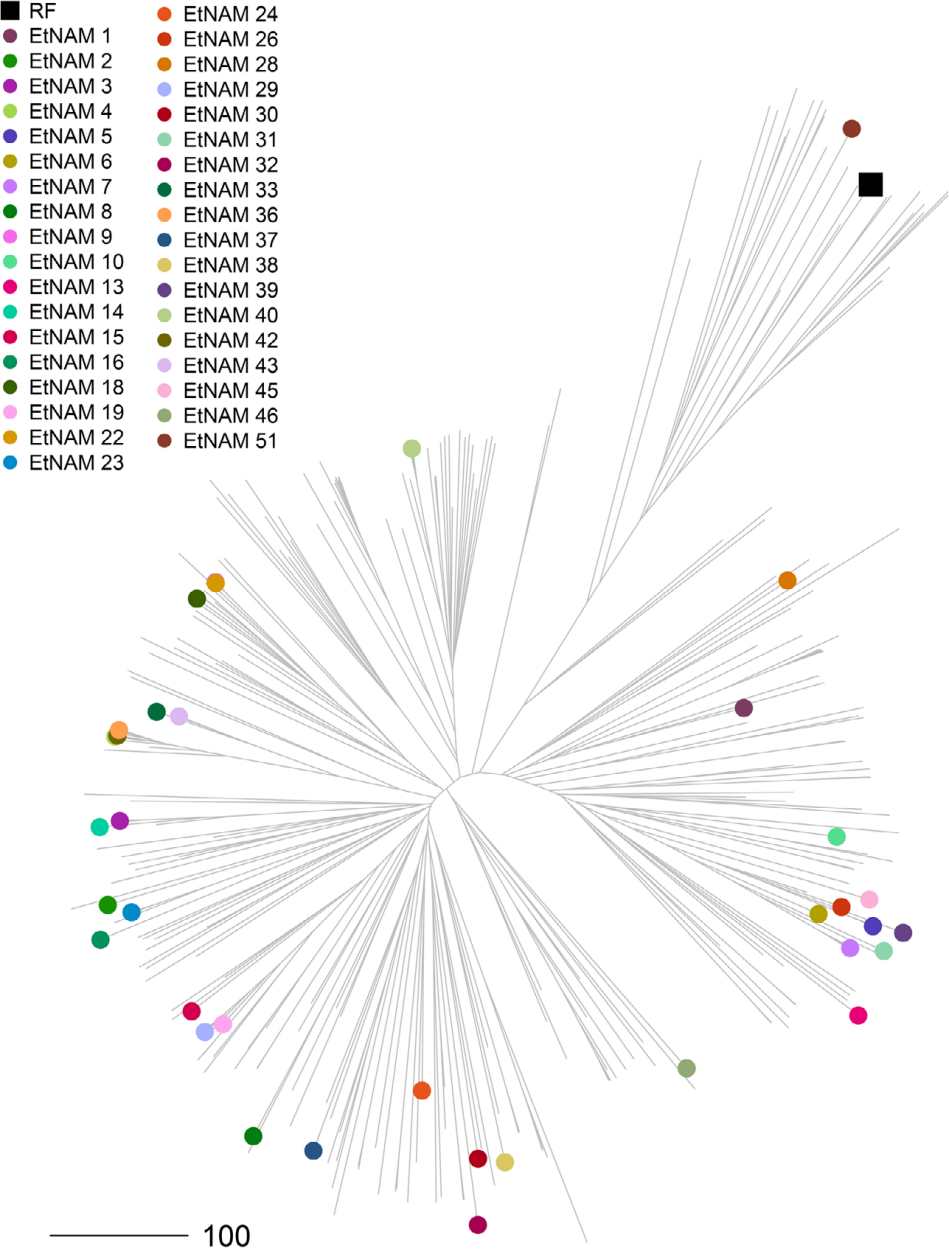
Molecular diversity of the EtNAM founder lines based on 30 155 SNPs reported in Mengistu *et al*. (2016). The NJ phylogeny represents the molecular diversity of Ethiopian durum wheat. Two main clades are present: the smaller clade of MVs (top right), and the larger clade of FVs (bottom left). EtNAM founder lines with a known genetic makeup are projected on the tree, and are represented as points coloured according to the legend. The recurrent founder (RF), Asassa, is represented by a dark square and groups together with the MV Bidi (EtNAM 51) in the monophyletic cluster top right. Data from Mengistu *et al*. (2016)

**Figure 2:**
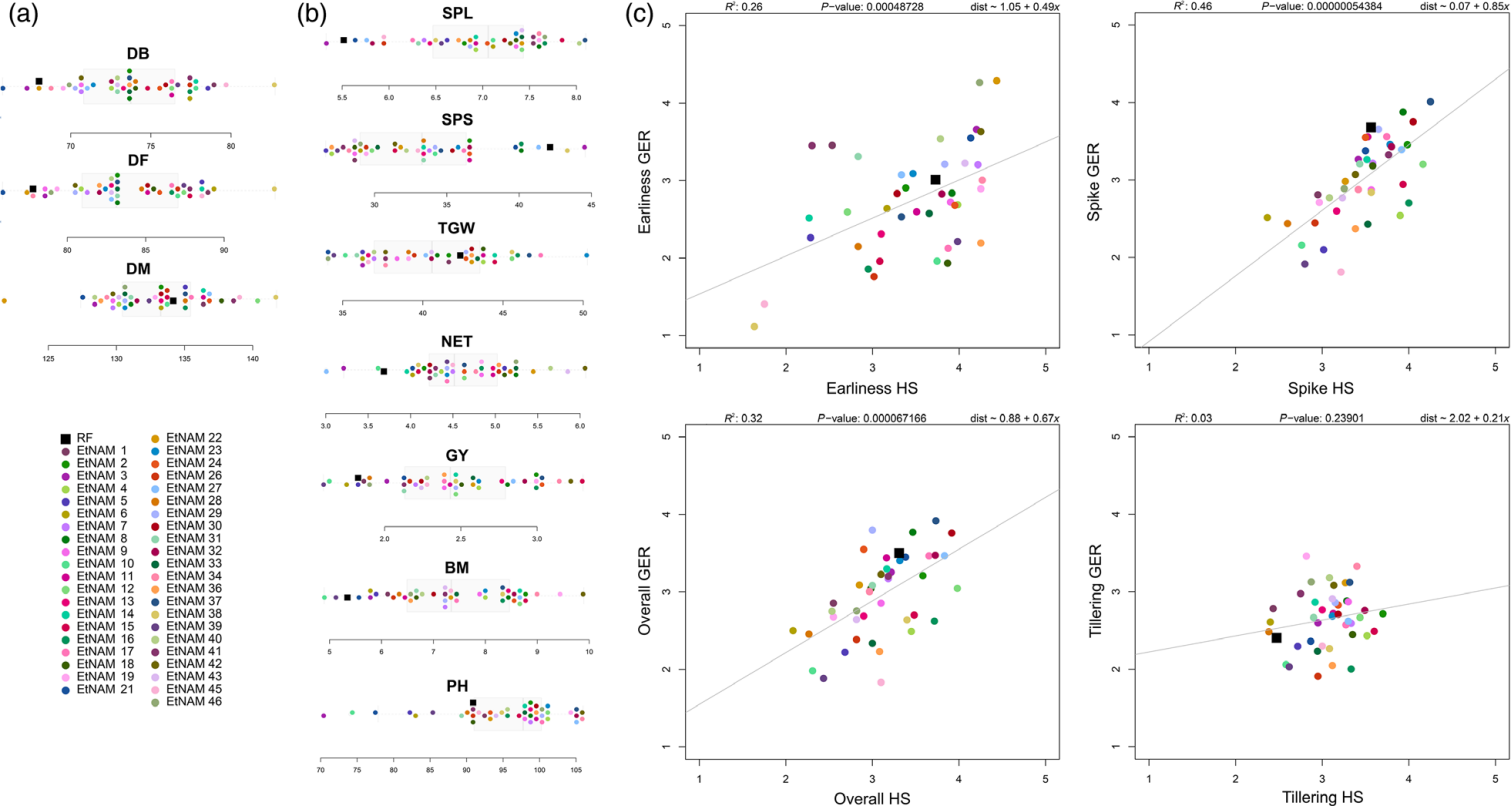
Phenotypic diversity of the EtNAM founder lines based on data reported in Mengistu *et al*. (2016), Kidane *et al*. (2017b) and Mancini *et al*. (2017). (a) Distribution of the EtNAM founder lines according to phenology traits (DB, days to booting; DF, days to flowering; DM, days to maturity). EtNAM founders are represented by points coloured according to the legend and are overlaid on a bar plot reporting the distribution of the trait. (b) Distribution of EtNAM founder lines according to agronomic traits, as depicted in panel (a) (SPL, spike length; SPS, number of seeds per spike; TGW, thousand grain weight; NET, number of effective tillers; GY, grain yield; BM, biomass; PH, plant height). (c) Distribution of EtNAM founder lines according to smallholder farmers’ appreciation measured in Geregera (Amhara, Ethiopia) and Hagreselam (Tigray, Ethiopia). Farmer traits are: Earliness, appreciation of flowering time; Spike, appreciation of the spike traits; Overall, general appreciation of the variety; Tillering, appreciation of tillering capacity. In each scatter plot, the *x*‐axis reports the value given by farmers in Hagreselam, and the *y*‐axis reports the value given by farmers in Geregera (from 1, low appreciation, to 5, high appreciation). A regression line is reported in gray in each plot. EtNAM founders are depicted as in panel (a). Details of the linear model run across locations are reported above each graph.

The EtNAM population is composed of a total of 6280 F_9_ RILs divided into 50 families (Table S1). One of the FV founder lines, with accession number 238555, was later discovered to be bread wheat. The corresponding EtNAM 49 family only produced 15 RILs; hence, it was excluded from the population. The EtNAM families range in size from 83 to 205 RILs (Table S1; mean = 125.6, σ = 28.2). During single seed descent (SSD), a broad variability was visible within and between families in terms of disease resistance, spike morphology, awn colour, plant height, and the length of the plant cycle. The amount of genotypic differentiation among EtNAM RILs is comparable to that among founder lines. Previous studies characterized the geographic, molecular, and phenotypic diversity of Ethiopian MVs and FVs, including the EtNAM founder lines (Mengistu *et al*., 2016). Figure 1 shows a phylogenetic tree based on 30 155 SNP markers reported in Mengistu *et al*. (2016). The EtNAM founders were selected to cover the broad molecular diversity of Ethiopian durum wheat and to provide a number of traits of breeding relevance such as spike density, yield, and disease resistance (Table S1). Consequently, the EtNAM founder lines showed elevated phenotypic variance for a number of traits relevant for Ethiopian agriculture (Table 1, Figure 2). Previously published data characterizing Ethiopian FVs shows that days to booting and flowering among EtNAM founders span more than 2 weeks (Mengistu *et al*., 2016). Most of Ethiopian FVs require fewer days to reach maturity than the recurrent founder (RF), the MV Asassa (Figure 2a). The RF contributes agronomic traits typical of international breeding targets for MVs (Figure 2b): spike length (SPL) is reduced, yet seeds per spike (SPS) are many, resulting in dense spikes. The thousand grain weight (TGW) is high, while the number of effective tillers (NET) produced and the plant height (PH) are low. Notably, most of the FV founders of the EtNAM perform better than the RF in the tested Ethiopian environments, both in terms of grain yield (GY) and biomass production (BM). The broad variation in agronomic traits among EtNAM founder lines (Table 1) ensures broad segregation of these traits in the EtNAM RILs. The EtNAM RF and FV founders were also selected considering smallholder farmers’ appreciation, which is measured by four traits evaluated on a scale from 1 to 5 as previously reported in Kidane *et al*. (2017b) and Mancini *et al*. (2017). The farmer communities in the two tested locations, Hagreselam and Geregera, showed high consistency in the evaluation of earliness capacity, spike morphology, and overall appreciation of the EtNAM founder lines (Figure 2c). Asassa was chosen as RF since it was among the most appreciated MVs in the tested locations. This preference was confirmed by farmer scores that consistently ranked the RF among the most valued genotypes, although several locally adapted FVs outperformed it (Figure 2c). Farmer traits span from less than two to more than three score points (Table 1) in both locations (Kidane *et al*., 2017b; Mancini *et al*., 2017), suggesting broad variation in smallholder farmers’ appreciation to be found in EtNAM RILs. The subset of RILs to be genotypically characterized, covering EtNAM families 1, 3, 5, 8, 10, 16, 19, 32, 36, 45, 46, and 51, was selected on the basis of phenotypic and genotypic diversity of the founder lines and number of RILs available (Table S1).

**Table 1:** Phenotypic values of the EtNAM founder lines based on data reported in Mengistu *et al*. (2016), Kidane *et al*. (2017b) and Mancini *et al*. (2017). For each trait, the table reports the minimum (Min), maximum (Max), and mean (Mean) value. Standard deviation (SD) and range (Range) are also reported. Farmer traits (Earl, Over, Spike, Tiller) are given in a scale going from 1 (worst) to 5 (best)

Phenotype	Min	Max	Mean	SD	Range
DB (days)	65.75	82.75	73.76	3.72	17.00
DF (days)	75.88	93.25	83.80	3.95	17.38
DM (days)	121.75	141.75	133.05	3.84	20.00
PH (cm)	70.38	105.96	95.00	8.24	35.58
NET (n)	3.00	6.07	4.60	0.64	3.06
SPL (cm)	5.33	8.10	6.93	0.70	2.77
SPS (n)	26.63	44.58	33.29	4.80	17.95
BM (t/ha)	4.90	9.90	7.37	1.24	5.00
GY (t/ha)	1.60	3.30	2.43	0.45	1.70
TGW (g)	34.08	50.26	40.59	4.03	16.19
Earl HS	1.63	4.43	3.47	0.72	2.80
Tiller HS	2.38	3.70	3.06	0.33	1.32
Spike HS	2.37	4.25	3.46	0.43	1.88
Over HS	2.08	3.98	3.11	0.46	1.90
Earl GER	1.12	4.29	2.75	0.69	3.17
Tiller GER	1.91	3.46	2.65	0.37	1.55
Spike GER	1.81	4.01	3.00	0.53	2.20
Over GER	1.83	3.92	2.96	0.53	2.08

BM, biomass; DB, days to booting; DF, days to flowering; DM, days to maturity; Earl GER, Spike GER, Tiller GER, and Over GER, earliness, spike, tillering, and overall appreciation in Geregera; Earl HS, Spike HS, Tiller HS, and Over HS, earliness, spike, tillering, and overall appreciation in Hagreselam; GY, grain yield; NET, number of effective tillers; PH, plant height; SPL, spike length; SPS, number of seeds per spike; TGW, thousand grain weight.

### Genetic diversity in the EtNAM subset

The genotyping array used to characterize the subset of EtNAM RILs investigated comprises 13 006 markers. After filtering for quality (Failure rate < 20% and Heterozygosity < 20%), 12 114 markers were retained. The EtNAM families in the subset have a number of polymorphic SNPs ranging from 1665 (EtNAM 51) to 4110 (EtNAM 3), with a mean of 3479 (Table S2). Family EtNAM 51, in which both parents are MVs (Asassa//Bidi), has a lower number of SNPs and low heterozygosity and failure rate (0.49% and 0.31%, respectively; Table S2). This is possibly contributed by ascertainment bias in the genotyping array, with some probes failing to effectively capture FV allelic variation while providing better performances on MV alleles. Although the MAF in individual families is close to 50%, with some low‐frequency alleles due to residual heterozygosity, the MAF in the EtNAM is broadly distributed (Figure S1). The segregation of rare alleles in the population allows a better representation of the broad diversity of Ethiopian wheat. The difference between EtNAM 51 and the other families is clear when looking at the intersection of polymorphic markers in the subset (Figure S1). The largest share of polymorphic markers in the population, 862, is shared only among families with Ethiopian FV founders. The second largest share of markers (216) is unique to EtNAM 51, and only 141 markers are polymorphic in all the EtNAM families characterized. The most diverse family, EtNAM 3, has the highest number of unique SNPs (102), followed by EtNAM 10 (79), and EtNAM 32 (74) (Figure S1).

**Figure 3:**
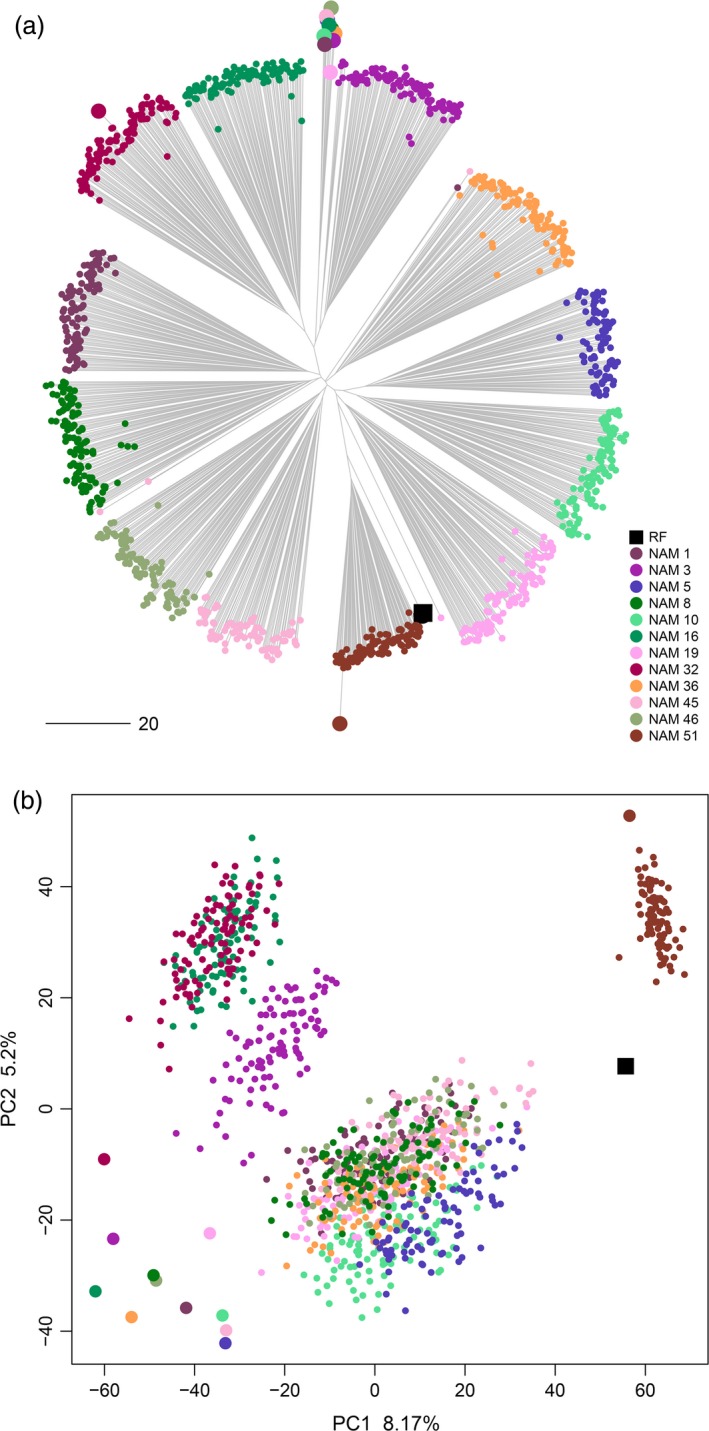
Molecular diversity of the EtNAM subset. (a) NJ phylogeny representing the genetic relationship across EtNAM RILs, coloured according to the EtNAM family of provenance as per the legend. EtNAM founder lines are represented by larger points following the same colouring scheme. (b) Outcome of PCA on genotyping data. PC1 and PC2 are shown on the *x*‐ and *y*‐axes, respectively, along with their explained variance. EtNAM RILs and founder lines are represented as in panel A.

The molecular diversity in the EtNAM subset overlaps the breeding scheme of the population (Figure 3). The MVs (the RF Asassa and the line Bidi) and the FV founders are the furthest apart in a neighbour‐joining (NJ) phylogeny. In the middle, the EtNAM RILs cluster into 12 monophyletic clades corresponding to each of the interconnected biparental families (Figure 3a). The signal of a few RILs grouping outside their intended families is likely resulting from either DNA or seed contamination (Figure 3a). The EtNAM RILs fill the gap between Ethiopian FVs and MVs in the first two principal components (PCs) computed on molecular diversity (Figure 3b). Expectedly, the MV founder of EtNAM 51 is opposite to the FVs, and EtNAM 51 RILs cluster intermediately next to the RF. The principal components analysis (PCA) provides new variables each explaining a little amount of variability, suggesting low structure in the population. The first two PCs explain 8% and 5% of the molecular variance, respectively, and the explanatory power of subsequent PCs drops rapidly, requiring 20 PCs to approach 50% of the molecular variance (Figure S2).

Following the filtering parameters listed in the Methods, 9383 SNP markers were physically mapped to the A and B genome of wild emmer wheat (Table S3). Diversity across the EtNAM genomes is sampled according to the array markers’ distribution on the wheat genome, which is reduced in pericentromeric regions. Surveying the genomic distribution of polymorphisms in the EtNAM subset, we do not observe regions of depleted diversity (Figure S3). Diversity within each EtNAM family is dependent on the allelic makeup of founder pairs. A discriminant analysis of principal components (DAPC) expectedly points to the presence of 12 genetic groups (Figure S4). The degree of differentiation among EtNAM families, however, is not uniform and results in three main clusters (Figure S4) whose differentiation is supported by a limited set of markers (Figure S5). The variance explained by the first component is mostly contributed by a set of markers in the distal portion of Chr 6A. The markers providing the higher contribution to the variance explained by the second component are located on the distal portions of Chr 1A and Chr 4A, while the variance explained by component three is mainly contributed by markers at the end of Chr 7A.

**Figure 4:**
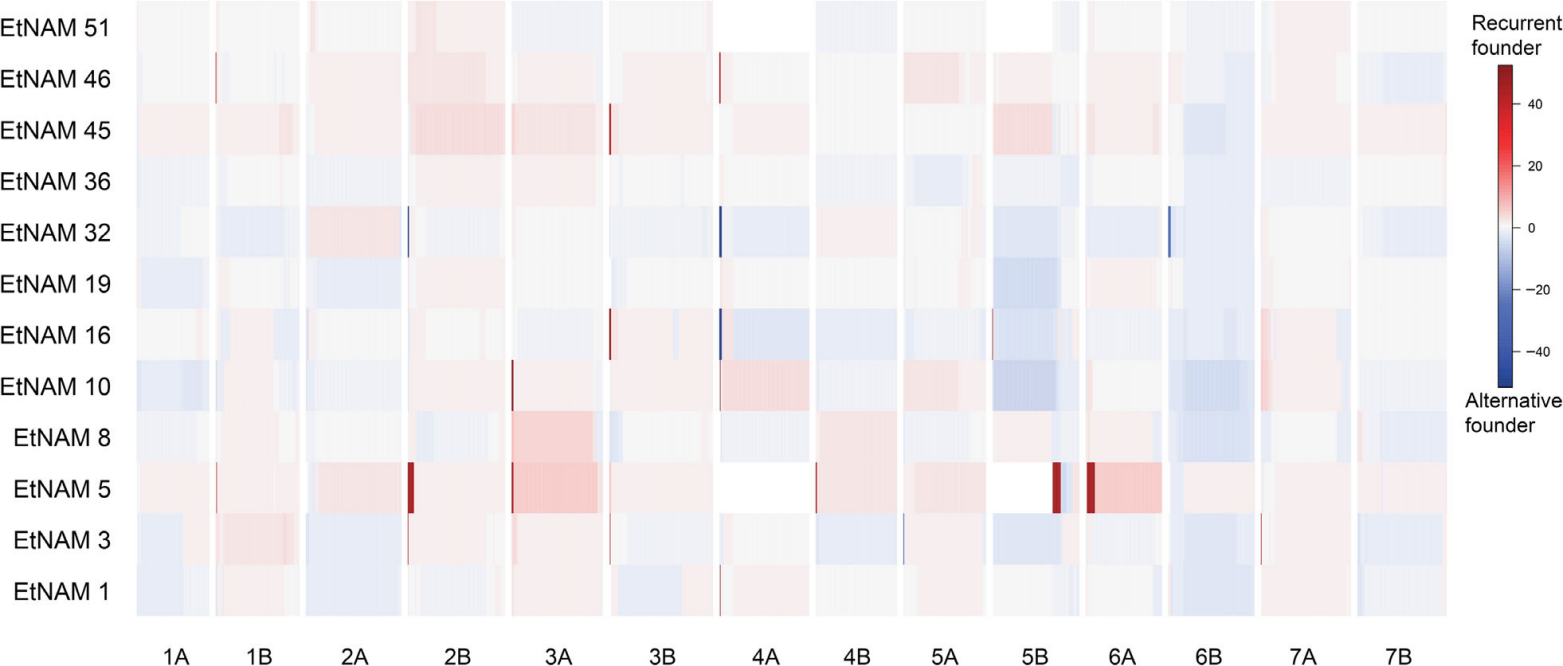
Deviation in founder contributions across EtNAM genomes. The binned genomic positions are reported on the *x*‐axis. The EtNAM families are reported on the *y*‐axis. The deviation from the expected founder contribution is represented by increasing shades of red (RF overrepresented) or blue (alternative founder overrepresented). The significance of the deviation is represented in negative log scores of chi‐squared test *P*‐values combined within each genomic bin. The threshold for significance is 13.

The RF contribution to RIL genomes is mostly uniform, with some localized genomic regions with biased representation of either of the founder alleles for some of the EtNAM families (Figure 4, Table S4). The significance threshold for combined negative log scores of *P*‐values, computed with a Bonferroni multiple test correction, is 13. Family EtNAM 5 presents the highest number and extension of such regions on Chr 2A, 2B, 3A, 4B, and extensively on Chr 5B and Chr 6A. Some regions of biased inheritance of parental allele are present in more than one EtNAM family, including regions at the beginning of Chr 2B (EtNAM 5, EtNAM 32), on Chr 3A (EtNAM 5, EtNAM 10), on Chr 3B (EtNAM 3, EtNAM 16, EtNAM 35), and on Chr 4A (EtNAM 1, EtNAM 10, EtNAM 16, EtNAM 32, EtNAM 46). In some of these genomic bins, the founder unbalance is contrasting among families, with some showing an overrepresentation of the RF and others showing overrepresentation of the alternative founder line (Figure 4).

**Figure 5:**
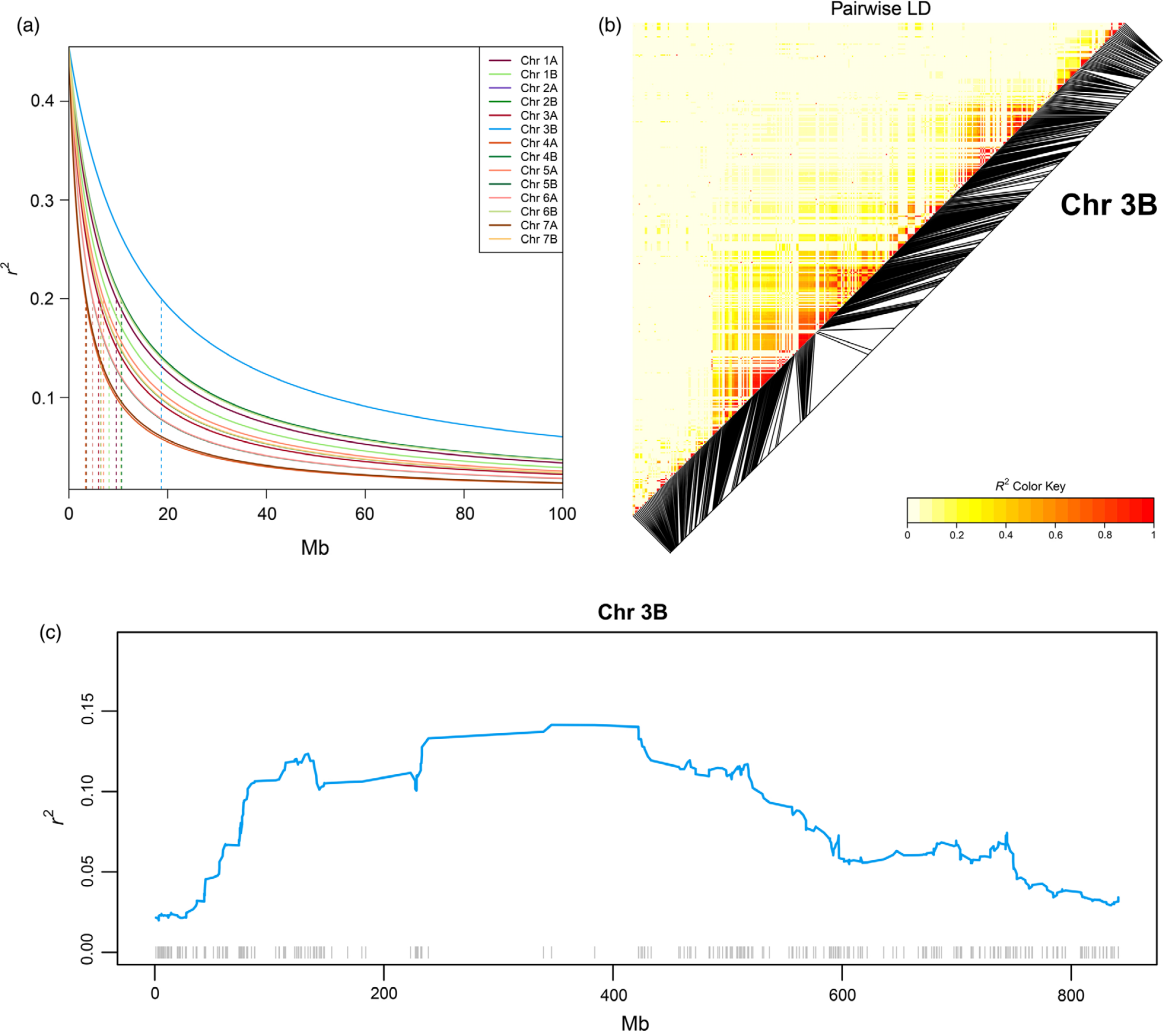
Linkage disequilibrium (LD) in the EtNAM subset. (a) LD (*y*‐axis) decay over physical distance (*x*‐axis) in each Chr, coloured according to the legend. The distance needed for a lack of LD (*r* ^2^ *= *0.2) is represented by vertical dashed lines for each Chr. (b) Pairwise LD measures on Chr 3B, the Chr with the slowest LD decay. Markers are ordered according to their physical positions, and black segments are projected onto their map position. Increasing values of *r* ^2^ are reported in increasing shades of red according to the legend. (c) LD evolution across Chr 3B. LD measures are averaged within genomic bins and represented as a continuous line coloured as per panel (a). The Mb positions across the Chr are shown on the *x*‐axis. Molecular markers available on Chr 3B are represented by grey ticks at the bottom of the plot in the corresponding physical positions.

The profile of linkage disequilibrium (LD) decay is different across EtNAM families, and Chr 3B, 4B, and 6B show the slowest decay in several of them (Figure S6). Overall, the *r* ^2^ LD drops below 0.2, an arbitrary threshold for lack of LD, within 7.4 Mb on average, ranging from 3.7 Mb (Chr 4A) to 18.7 Mb (Chr 3B) (Figure 5a). Pericentromeric regions show higher LD, as expected from the lower recombination rate, balancing reduced LD in Chr telomeres (Figure 5b and Figure S7). The estimation of LD is dependent on the availability of marker information, which is sparser across pericentromeric regions (Figure 5C and Figure S3). Areas of relatively high LD may also be found in telomeric regions, notably at approximately 650 Mb on Chr 2B, 520 Mb on Chr 3A, 130 Mb on Chr 6B, and 600 Mb on Chr 7A (Figure S8).

### Genome wide association study

**Figure 6:**
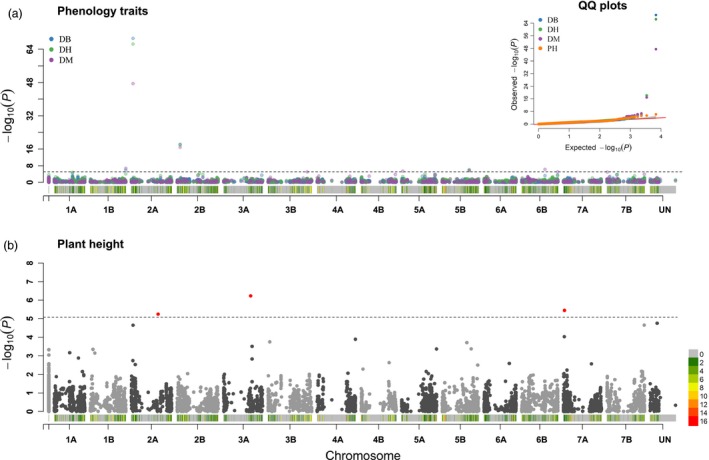
Outcome of the GWA analysis on the EtNAM. (a) Manhattan plot overlapping the GWA scans results on DB, DH, and DM with colours according to legend top left. On the x axis, the physical position of tested markers with a representation of SNP density in colours according to the scale bottom right. On the y axis, the significance of each association reported in negative logarithm of *P* values. The threshold represents a Bonferroni correction for a nominal *P *<* *0.1. Markers surpassing the threshold (dashed black line) are QTNs. The insert top right represents the QQ plot for all tested traits. (b) Manhattan plot resulting from the GWA for PH, represented as in panel (a). QTNs are highlighted in red.

To provide a benchmark of its potential in complex traits mapping, the EtNAM subset was phenotyped in three locations for phenology (days to booting, DB; days to heading, DH; days to maturity, DM) and plant height (PH) (Figure S9). The broad – sense heritability (h^2^) was 90.1% for DB, 89.5% for DH, 60.8% for DM and 59.5% for PH. Best linear unbiased prediction (BLUP) values were derived from trait values and were used to conduct a GWA study to identify quantitate trait nucleotides (QTNs), *i.e*. SNPs in linkage with causal variants underlying the trait variation in the EtNAM (Table S5). The EtNAM population reported 11 unique QTNs significant at a *P *<* *0.1 Bonferroni corrected significance treshold (Table 2, Table S5). QTNs for DB, DH, and DM were partially overlapping (Figure 6a): QTNs at 35.8 Mb on Chr 2A and 520.6 Mb on Chr 5B were jointly identified by all phenology traits. A QTN for DB and DH mapped at 57.2 Mb on Chr 2B, and two QTNs for DB and DM mapped at 677.9 and 679.8 Mb on Chr 1B. Additional QTNs for DM mapped at 56.7 Mb on Chr 2B, at 19.9 Mb on Chr 5A, and at 447.3 Mb on Chr 6B. PH identified three QTNs mapping at 514.3 Mb on Chr 2A, 516.7 Mb on Chr 3A, and 16.9 Mb on Chr 7A (Figure 6b, Table 2). Altogether, the GWA scan identified 177 unique protein‐coding gene models within 1 Mb of QTNs (Table S6). PH QTNs had an estimated effect ranging from 0.95 to 1.66 cm, while flowering time QTNs explained up to 3 days in booting difference and 1.9 days in maturity (Table 2).

**Table 2:** QTN identified on the EtNAM subset according to a Bonferroni correction of a nominal test *P *<* *0.1. For each QTN are specified the traits, the lead marker, the position according to *T. dicoccoides* coordinates and the effect estimated by the model (ordered according to traits in the first column)

Traits	Marker	Chromosome	Position	Effect
DB	wsnp_Ku_c13952_22097856	1B	677 945 596	−0.66
DM	Excalibur_c25640_110	1B	679 781 387	0.57
DB, DH, DM	BobWhite_c26374_339	2A	35 796 146	−3.04; −3.01; −1.91
PH	BobWhite_c46028_206	2A	514 332 256	0.95
DM	Kukri_c26288_419	2B	56 658 355	1.05
DB, DH	wsnp_Ex_c4349_7841003	2B	57 179 677	1.35; 1.36
PH	wsnp_Ex_c10667_17387885	3A	516 665 256	−1.66
DM	wsnp_Ex_c807_1585614	5A	19 874 811	−0.53
DB, DH, DM	Ra_c1726_1071	5B	520 597 193	0.78; 0.81; 0.51
DM	Excalibur_c15335_197	6B	447 303 008	0.73
PH	RAC875_c16644_491	7A	16 943 438	−1.6

DB: days to booting; DH: days to heading; DM: days to maturity; PH: plant height.

## Discussion

### Diversity in the EtNAM population

The founder lines contribute with broad phenotypic and molecular diversity to the EtNAM population. Having been chosen from the most divergent genotypes of Ethiopian FVs (Figure 1), they cover the spectrum of diversity available in local germplasm (Mengistu *et al*., 2016). Indeed, the EtNAM founders show a broad range of variability for several agronomic traits and for smallholder farmers appreciation (Figure 2, Table 1). As expected, the RF and MV included in family EtNAM 51 are the most similar and show trait values reminiscent of the Green Revolution: a short life cycle with a longer grain filling stage up to maturity (Figure 2a), as well as short stature and compact spikes coupled with low tillering capacity (Figure 2b). Notably, the MV genetic background does not provide higher yields in the marginal environments in which the EtNAM founders were measured. As per farmer appreciation traits (Figure 2c), the RF Asassa is consistently grouped among the most preferred genotypes in both locations, but it is surpassed by some of the FVs. It has been shown that phenotypic divergence among NAM founder lines is predictive of phenotypic variance in RIL families (Hung *et al*., 2012); hence, we expect broad variation for multigenic traits in EtNAM RILs.

The EtNAM subset was chosen to provide a balanced representation of the EtNAM population (Figure 1); hence the findings developed based on the 1200 RILs comprising the subset can reasonably be extended to the full EtNAM. The population is highly diverse and features a limited genetic structure (Figure 3 and Figure S2), which supports the selection of a subset of EtNAM families to target specific loci without losing the overall features of the population. However, the current picture of diversity among RILs and EtNAM families may be slightly biased by the genotyping technology employed. The markers featured on the array, in fact, are mostly designed based on international breeding germplasm (Wang *et al*., 2014) and may fail to capture the full spectrum of *exotic* alleles available in Ethiopian FVs (Albrechtsen *et al*., 2010). This hypothesis is supported by consistently lower levels of heterozygosity and a low failure rate in RILs developed from the cross between RF and MV in family EtNAM 51 (Table S2). It is therefore likely that the diversity contributed by FVs is underestimated, and that the EtNAM families with most diverse founders would cluster further away from each other (Figure 3a). The panel of polymorphic SNPs is quite differentiated between family EtNAM 51 and the other families (Figure S1). Most SNPs are shared across EtNAM families derived from FVs, with some families showing remarkable uniqueness. Indeed, DAPC analysis shows that EtNAM families cluster according to each founder's genomic makeup (Figure S4), with a relevant contribution of loci possibly selected during FV cultivation (Figure S5). However, it seems that DAPC grouping does not reflect the geographic origin of EtNAM founders (Table S1), in agreement with the high admixture observed within and between regional collections of Ethiopian FVs (Mengistu *et al*., 2016). Once additional EtNAM families are characterized, a more complete pattern of diversity and relatedness within the population will emerge.

It is possible that during the breeding process some traits have been inadvertently selected, resulting in a biased representation of founder lines in the RIL genomes (Figure 4). Similar deviations may be explained by the presence of QTL that provide an increase in fitness during the production of the population (Dell'Acqua *et al*., 2015; McMullen *et al*., 2009). Markers’ ascertainment bias may contribute to extended chromosomal regions in which no polymorphic markers are available, most notably on Chr 4A and 5B (Figure 4). A major QTL for heading date is located at approximately 158 cM on Chr 5B of winter wheat (Griffiths *et al*., 2009; Zanke *et al*., 2014a), and is in a position corresponding to markers mapping to approximately 690 Mb on the wild emmer genome (Table S3) and compatible with the overrepresentation of RF alleles on Chr 5B in EtNAM 5 (Figure 4, Table S4). Flowering time QTL are also located at initial positions on Chr 2A and 2B (Griffiths *et al*., 2009; Hanocq *et al*., 2004; Zanke *et al*., 2014a), also biased towards the RF in EtNAM 5. This interpretation is corroborated by the flowering time QTNs identified by the EtNAM on the on the homoeologous groups 2 and 5 (Figure 6, Table 2). The FV founder of EtNAM 5 shows remarkably late booting and flowering, which is quite opposite the RF (Figure 2a), and its late flowering alleles may have been selected against during the development of the EtNAM population. We detected a region at the beginning of Chr 4A in which the RF is either overrepresented or underrepresented in different EtNAM families (Figure 4). In this region several QTL have been mapped for seedling vigour (Zhang *et al*., 2013), seed production traits (Lv *et al*., 2014), and seed filling and senescence (Xie *et al*., 2016). Alternative alleles at this locus may affect fitness, and hence may have been subjected to selection during population development.

### The EtNAM as a QTL mapping and pre‐breeding tool

The NAM design provides an elevated recombination frequency and high QTL mapping power (Yu *et al*., 2008). The MAF distribution in the EtNAM population shows that the population captures rare alleles and sets them at higher frequencies (Figure 3a). It has been shown that joint‐multiple family is a powerful QTL mapping approach even in the presence of high heterogeneity between NAM families (Ogut *et al*., 2015), even though multiple QTL mapping approaches may be needed to capture the full extent of QTL effects in NAM‐like designs (Garin *et al*., 2017).

Thanks to the accumulation of recombination events across families, the LD decays much more rapidly in the EtNAM population (Figure 5a) than in any of the bi‐parental EtNAM families that comprise it (Figure S6). This finding supports the use of a multiparental population for high‐definition QTL mapping. It is important to point out that the capacity of markers to distinguish between paternal haplotypes may have an effect on the LD decay rate estimation. In some instances, in families EtNAM 5, 32, 36, and 46, the LD on Chr 5B and 6B extends for far more than 150 Mb (Figure S6). This is either because of linkage drag as a consequence of selection or because the typed markers show low polymorphism on these linkage groups in these families. When considering the full population, Chr 3B shows a somewhat higher LD and may be used as an example of the LD features of the EtNAM (Figure 5). As expected, pairwise LD is consistently higher in peri‐centromeric regions (Figure 5b), even though markers are preferentially located in telomeric regions (Figure S7). These results are in agreement with the recombination frequency distribution observed in a bread wheat NAM (Jordan *et al*., 2018). It is likely that the areas of high LD around centromeres are more extensive then what is apparent using the current marker set. The estimation of LD evolution indeed shows extensive regions with low recombination rates along peri‐centromeric regions (Figure 5c and Figure S8). The sparse distribution of array markers, which preferentially target coding regions (Figure S3), makes it difficult to finely characterize the extended peri‐centromeric regions of the wheat genome (Avni *et al*., 2017; The International Wheat Genome Sequencing Consortium, 2018).

Already with this marker set, however, we could identify 11 QTNs with relevant effects on phenology and plant height (Figure 6, Table 2). Some of the flowering time QTNs may correspond to previously reported flowering time loci segregating in European germplasm, even though the different genetic materials and reference maps limit the possibility to cross‐reference results. The study from Mengistu *et al*. (2016), conducting a GWA on landraces including the EtNAM founders reinforces some of these findings. The DB and DM QTNs on Chr 1B are compatible with previous findings on bread wheat (Fowler *et al*., 2016). The *Eps* locus controlling flowering time in bread wheat maps on Chr 1D (Sukumaran *et al*., 2016), at a genetic position overlapping our signal for DB, DM on the homoeologous Chr 1B (Table 2). QTL for flowering time were already reported in durum wheat at around 40 cM on Chr 2A, in a position compatible to our signal (Maccaferri *et al*., 2008; Mengistu *et al*., 2016; Sanna *et al*., 2014; Sukumaran *et al*., 2018). This QTN on Chr 2A maps in a position compatible to the *Ppd‐A1* locus (Griffiths *et al*., 2009). The flowering time QTN at about 57 Mb on Chr 2B (Table 2) may also correspond to previously reported QTL (Sanna *et al*., 2014; Sukumaran *et al*., 2018; Zhou *et al*., 2016) and may contribute to bias in founders’ contribution in the EtNAM RILs (Figure 4). The *Vrn‐A1* locus regulating the transition from vegetative to reproductive development is located on the homoeologous group 5 (Yan *et al*., 2003; Zhu *et al*., 2014), in a position compatible with the EtNAM QTN for DB, DH, DM (Table 2). Expectedly, phenology QTNs detected by the EtNAM were also detected in Ethiopian durum wheat landraces on Chr 1B (DM) and Chr 2B (DB, DH) (Table 2) (Kidane *et al*., 2017b). Previous GWA studies on spring wheat and durum wheat support the phenology QTN identified on Chr 2B (Soriano *et al*., 2017; Sukumaran *et al*., 2015). The signal for PH on Chr 2A and Chr 3A may also correspond to previously mapped QTL (Cui *et al*., 2011; Maccaferri *et al*., 2008; Zanke *et al*., 2014b). Notably, the PH QTN on Chr 2A matches a GWA signal for PH reported in durum wheat grown in water stress conditions but not in yield potential conditions (Sukumaran *et al*., 2018). A PH locus was already reported on the bread wheat Chr 7A in a position compatible to our findings (Fowler *et al*., 2016). The *TaHd1* genes, orthologous to the heading date genes *Hd1* in rice, were mapped on the long arm of the homologous group 6 (Nemoto *et al*., 2003), and may contribute to the signal we detected at the distal end of this chromosome. QTL controlling flowering time and frost resistance were mapped on the long arm of Chr 5B (Tóth *et al*., 2003). Our signal may be in the vicinity to *Vrn‐B1* locus (Leonova *et al*., 2003), whose role in wheat flowering is well known (Iwaki *et al*., 2002; Shimada *et al*., 2009; Yan *et al*., 2003). For the sakes of this experiment, we chose a stringent significance threshold to focus on highly significant QTNs, increasing type II errors; however, we expect several other alleles to contribute to these traits in the EtNAM.

Currently, the LD extent (Figure S6) and the sparsity of markers (Figure S3) on the EtNAM genomes prevent to pinpoint candidate genes underlying QTNs detected by GWA. As the genome annotation of wild emmer wheat will evolve, being further integrated with the functional analysis tools already available in other cereal species, it will be possible to provide a finer characterization of gene models in the vicinity of QTNs (Table S6). Moreover, once a genetic map specific to the EtNAM will be developed, it will be possible characterize QTNs in relation to founder haplotypes, supporting the identification of candidate genes. Indeed, genetic maps currently available in durum (Maccaferri *et al*., 2015) and bread wheat (Wang *et al*., 2014) focus on international breeding materials and Mediterranean landraces. The uniqueness of Ethiopian germplasm (Kabbaj *et al*., 2017; Mengistu *et al*., 2016) pushes for a specific genetic map to be developed in order to capture possible structural variants, a frequent occurrence in the highly repetitive wheat genome (Clavijo *et al*., 2017; Montenegro *et al*., 2017). We can anticipate that a genetic map is being produced on the EtNAM, and that such map will be used to survey the genomic landscape of Ethiopian durum wheat siding the upcoming high‐quality durum wheat genome sequence. These tools will boost the usability of sequencing‐based markers (Baird *et al*., 2008), allowing to overcome ascertainment bias issues introduced by array‐based genotyping, and support haplotype reconstruction and QTL mapping on the EtNAM.

The EtNAM will side the multiparental populations available (Milner *et al*., 2016; Huang *et al*., 2012; Jordan *et al*., 2018; Mackay *et al*., 2014) and those in development in the *Triticum* species complex. It has been shown that the parallel employment of NAM families developed in closely related species can reinforce QTL findings (Mace *et al*., 2013), increasing their potential use in breeding efforts. Unlike the maize NAM, where the RF is a standard breeding genotype with a sequenced genome (McMullen *et al*., 2009), the EtNAM RF was chosen because of its breeding potential in terms of Ethiopian farmers’ appreciation among MVs (Figure 2c). The EtNAM strategy is indeed aimed at contributing to new recombination between international and Ethiopian material, leveraging local diversity in combination with *elite* germplasm. The characterized EtNAM RILs successfully close the gap existing between the two allelic pools (Figure 3) and arguably recombine traits contributed by either of the founder lines. The use of FVs and landraces in breeding has long been advocated to reverse the trend of diversity loss resulting from the early Green Revolution (Lopes *et al*., 2015; Sehgal *et al*., 2015; Warburton *et al*., 2006). In Ethiopia, the higher yielding and performing bread wheat varieties imported from abroad are rapidly replacing local germplasm (Mengistu *et al*., 2015; Tsegaye and Berg, 2007). Improved varieties of durum wheat are rarely grown because of a lack of adaptability and low access to seeds (Tesemma and Bechere, 1998). The phenotypic traits included in the EtNAM (Table S1) are promising in providing raw material for the development of new durum wheat varieties with an optimal balance between MV and FV alleles for the benefit of breeding in Ethiopia and beyond.

## Experimental procedures

### Choice of founder lines and phenotypic characterization

The choice of the founder lines derives from the extensive molecular and phenotypic characterization of a core collection of Ethiopian FVs and MVs (Mengistu *et al*., 2016, 2018). FVs were obtained from the *ex situ* collection of Ethiopian germplasm at the Ethiopian Biodiversity Institute (EBI, www.ebi.gov.et). The accessions deriving from the EBI (5 g each) were purified during 2011, selecting one representative spike each (Mengistu *et al*., 2016). The selected spikes were selfed in well‐spaced plots to control for the already minimal outbreeding propensity of wheat. The EtNAM founders data used in this study as well as the spikes used to produce the EtNAM population derive from such single spike. The EtNAM founders were chosen with the criteria of (i) maximizing phenotypic and genotypic diversity among FVs and (ii) incorporating traits of interest, such as disease resistance, drought tolerance, and yield capacity as observed in the field trials of the collection. Genotypic data and phenotypic data of EtNAM founders lines may be found in Mengistu *et al*. (2016). Farmer appreciation data may be found in Kidane *et al*. (2017b) and Mancini *et al*. (2017). Fifty such FVs were chosen, 49 of which are currently represented in the EtNAM population. One additional MV with an Italian pedigree, Bidi, was also chosen as a founder. The RF, Asassa, was chosen because it was the best performing and most appreciated by farmers in testing locations of the core collection. Asassa is an MV with CIMMYT background that was released in Ethiopia in 1997 by the Ethiopian Institute of Agricultural Research (EIAR). The Asassa pedigree is the following: CHO/TARUS//YAV/3/FG/CRA/5/FG/DOM/6/HUI or CHORLITO/YAVAROS//FREE‐GALLIPOLI/3/FREE‐GALLIPOLI/CANADIAN‐RED/4/FREE‐GALLIPOLI/DON‐PEDRO/5/HUITLE. The FV founders were collected largely from the Amhara and the Oromia regions with a few from the Tigray region of Ethiopia. The EtNAM founders retained in the current population, listed with their passport data and traits of interest, are reported in Table S1.

### Production of the EtNAM population

F_1_ crosses were produced in 2012 using Asassa, the RF, as pollen donor. We used a single spike from each FV female parent to start the crossing. For each female, we crossed five spikes with the RF. For each F_1_ (accessions #1 to #51× RF), F_2_ progenies were produced by subsequent selfing of F_1_ plants under controlled conditions during 2013. Four additional cycles of controlled selfing were carried out starting with 252 single plants per F_2_ family and eventually producing RIL‐F_6_ in 2015 by growing two cycles per year when possible. During the inbreeding phase we followed an SSD approach by harvesting only the main tiller and planting only three seeds per line starting from F_3_. EtNAM RILs were coded with an increasing number from 1 to 252 attached to the NAM family number (*i.e*., from EtNAM1_1 to EtNAM50_252). There are currently gaps in the numeric series corresponding to genotypes lost during the SSD procedure due to infertility or stochastic reasons. The data reported in this manuscript are based on the F_6_ generation. When this manuscript was written, the EtNAM was at the RIL‐F_9_ stage. All the crosses were conducted at the Sirinka Agricultural Research Center, Sirinka, Ethiopia. The EtNAM lines are stored at the Amhara Regional Agricultural Research Institute (Ethiopia) and are available to researchers upon signing a material transfer agreement, free of charge for public institutions.

### Selection and genotyping of the EtNAM subset

The EtNAM comprises 6280 RILs. A subset of EtNAM families was selected to provide an initial characterization of the population. Twelve EtNAM families were selected because of genetic diversity, traits of interest, and RILs availability. For each of the 12 EtNAM families in the subset, 100 RILs were germinated in an open field, five seedlings each, at the Sirinka Agricultural Research Center, Sirinka, Ethiopia. When they reached sufficient biomass, seedlings for each RIL were harvested and pooled in equal amounts. Green tissues were oven‐dried at 45°C for 72 h, and later maintained in sealed bags with silica gel beads (Sigma‐Aldrich, St Louis, MO). Samples were shipped to Italy and genomic DNA was extracted with the GenElute Plant Genomic DNA Miniprep Kit (Sigma‐Aldrich, St Louis, MO) following the manufacturer's instructions. DNA was checked for quantity and quality by electrophoresis on a 1% agarose gel and NanoDrop 2000 (Thermo Fisher Scientific Inc., Waltham, MA). Genotyping was performed on the Infinium 15K Ultra HD chips, derived by subsampling a set of markers optimized for durum wheat from the wheat 90K SNP chip (Wang *et al*., 2014) and performed at TraitGenetics GmbH (Gatersleben, Germany) as a service. SNPs were called using the tetraploid wheat pipeline in GenomeStudio V11 (Illumina, Inc., San Diego, CA) at the genotyping facility with default parameters.

### EtNAM founder data analysis

All data filtering and diversity analyses were conducted with custom scripts in R (R Core Team, 2018), available at https://doi.org/10.6084/m9.figshare.6304400. The EtNAM founder lines were genotyped with a core collection of Ethiopian FVs using the wheat 90K genotyping array (Wang *et al*., 2014), full results are presented in Mengistu *et al*. (2016). In this study, EtNAM founder data were checked for quality and consistency, and SNP markers with failure rate greater than 20% and heterozygosity above 20% were removed. An NJ tree was computed in R/adegenet (Jombart, 2008) to put in relation the EtNAM founder lines with other Ethiopian FVs and MVs. Phenotypic data for the EtNAM founder lines were derived from Mengistu *et al*. (2016) and plotted with R/beeswarm (Eklund, 2016). Phenotypic data of the EtNAM founder lines were collected at two locations in Ethiopia, Hagreselam and Geregera, during the 2012 and 2013 seasons. Full phenotypic data analysis is reported in Mengistu *et al*. (2016). Farmer trait data were collected by 30 smallholder farmers in each location during the 2012 season. Farmers were asked to rate each genotype from 1 to 5 for four traits of their interest: earliness, spike morphology, tillering capacity, and overall appreciation. Details on farmers’ data collection and analysis can be found in Mancini *et al*. (2017).

### EtNAM subset data analysis

Genotypic data produced for the EtNAM RILs were filtered for quality by removing unreliable samples and markers. Samples with less than 80% genotyped SNPs or with more than 20% heterozygous SNPs were removed from further analyses. SNP markers genotyped in < 80% of samples and those heterozygous in more than 20% of samples were subsequently removed. The filtered samples were sorted by family, and each family was individually checked for the presence of outlier samples when computing NJ phylogenies with R/adegenet (Jombart, 2008). Family clusters were individually inspected for clearly misassigned samples, and these samples were removed from further analyses as they were likely due to contamination at the seed lot or at the DNA extraction level. The remaining sample set of with high‐quality markers, was used to compute marker statistics within families and in the EtNAM population as a whole with R/adegenet (Jombart, 2008). Intersecting sets of markers among families were computed and visualized with R/UpSet (Conway *et al*., 2017). An NJ phylogeny was computed based on the EtNAM diversity data as a whole, and a PCA was used to evaluate the structure existing within the EtNAM subset.

The physical position of each SNP marker was derived from the wild emmer wheat genome assembly (Avni *et al*., 2017). The nucleotide sequence upstream and downstream of each SNP marker was derived from the array data. Sequences were considered single‐end reads, and converted in fastq format using a Perl script publicly available at https://github.com/ekg/fasta-to-fastq. The synthetic reads obtained were mapped onto the wild emmer genome with default parameters using bwa mem (Li, 2013). Samtools (Li *et al*., 2009) was used to filter reads with low mapping quality (MAPQ<10), secondary alignments, and multiple hits on the target genome. In this manuscript, we refer to wild emmer chromosomes as durum wheat's.

Individual RILs were clustered with a DAPC implemented in R/adegenet (Jombart, 2008) to identify cryptic similarities between EtNAM families. A clustering algorithm based on Bayesian information criteria (BIC) was run on RIL data, and the three main DA dimensions derived from the DAPC were used to compute individual loadings among markers having a physical position, thus deriving information of genomic loci maximizing the uniqueness of EtNAM families. The genomic distribution of the polymorphisms was compared with marker density by counting the occurrence of SNPs in genomic bins of 10 Mb. In each bin, the number of polymorphic markers was compared to the number of available markers.

The genomic contribution of the RF was computed independently within each EtNAM family. For each founder pair, all heterozygous markers were set as failed, and monomorphic markers and markers typed in only one of the founders were filtered out. The remaining markers, all polymorphic for founder alleles, were checked for distortion in their representation in the RILs, which was expected to be 50% for each founder. A chi‐squared test for goodness‐of‐fit was computed at each locus, and the resulting *P*‐values were averaged in 5 Mb intervals and log‐transformed to provide a measure of statistical significance. Only intervals with more than two markers were used to report *P*‐values. A Bonferroni threshold for the nominal *P*‐value of 0.05 divided by the number of markers in the population was used for significance. To provide an informative representation of the alternative parental contribution across the genome, the *P*‐values were multiplied by 1 if the RF allele was more frequent and by −1 if the alternative allele was over‐represented.

Pairwise LD was calculated for all markers with an assigned physical position on each Chr. R/LDheatmap (Shin *et al*., 2006) was used to compute *r* ^2^ within each Chr. The Chr‐specific LD decay rate was estimated on the basis of the Hill and Weir equation (Hill and Weir, 1988; Marroni *et al*., 2011) and discussed considering *r* ^2^ = 0.2 as a measure of a lack of LD. The LD evolution across each Chr was estimated by collapsing pairwise LD measures on the physical positions of markers. For each marker, the LD was averaged across all markers within a 0.5 Mb interval. The resulting LD was plotted across Chr averaging in a rolling window with a window size of 30 markers.

### EtNAM subset phenotyping and GWA

The EtNAM subset was phenotyped using a replicated alpha lattice design in three locations in Ethiopia: Adet (11°15′N/37°29′E) in 2016, Geregera (11°40′N/38°52′E) in 2016, and Kulumsa (8°01′N/39°09′E) in 2017. Seed rate of 100 kg/ha and fertilizers rate of 92 N + 46 P_2_O_5_ kg/ha were used in all locations. Urea was applied at a rate of 150 kg/ha using a split application of 1/2 at planting and 1/2; at tillering, and manual hand weeding was applied as required. Phenology traits were recorded on full plots: DB (days to 50% booting), DH (days to 50% heading), and DM (days to full maturity). PH was measured on three random plants per plot, in cm. BLUPs were calculated for all traits with restricted maximum likelihood (REML) analysis using SAS ^®^ (SAS Institute Inc 2013, Cary, NC). A mixed model describing the variate *Y*_*ijk*_ from replication *i,* column *j*, and row *k*, can be expressed by the following equation (Eq. 1):

(1) $$Y_{\mathrm{ijk}}=\mu +Ga+Lb+GLab+b_{i}+c_{j}+r_{k}+\epsilon_{\mathrm{ijk}}$$

where the fixed model effects are: μ, the intercept; *Ga*, the genotype main effect; *Lb*, the locations main effect; *GLab*, the interaction effect between genotypes and locations. The random model terms are: *b*_*i*_, the effect of replication *b*;* c*_*j*_ *,* the effect of columns within replication *b*;* r*_*k*_, the effect of rows within replication *b* and *∈*_*ijk*_, the overall error terms. The overall error term was estimated from the random terms of replications, rows and columns where rows and column were nested in replication. *h* ^2^ across the three locations was calculated from variance estimated for each component by REML analysis in SAS ^®^ (SAS Institute Inc 2013, Cary, NC) according to Vargas *et al*. (2013).

Trait values and SNPs were input in a GWA analysis using R/MVP (available at *github.com/XiaoleiLiuBio/MVP*) with custom scripts. The estimation of variance components was performed using the EMMA method (Kang *et al*., 2008), and mapping was done with the FarmCPU approach (Liu *et al*., 2016) including three PC covariates. The threshold for significance was corrected for multiple testing using a Bonferroni cut‐off on a nominal *P* value of 0.1. Associations surpassing the threshold are discussed as QTNs, *i.e*. SNPs in linkage with causative variants of quantitative traits. Gene models within ± 500 Kb from QTN were derived from the wild emmer genome annotation v1.0.59.

## Conflict of interest

The authors declare no conflicts of interest.

## Supporting information


**Figure S1** Features of the marker data produced in the EtNAM subset.

**Figure S2** Principal component analysis of molecular diversity in the EtNAM subset.

**Figure S3** Distribution of markers and SNPs in the EtNAM subset.

**Figure S4** Structure in the EtNAM subset.

**Figure S5** Marker loadings deriving from the DAPC.

**Figure S6** Chromosome‐specific linkage disequilibrium (LD) decay as a function of physical distance in each of the EtNAM families.

**Figure S7** Pairwise linkage disequilibrium (LD) in the EtNAM subset.

**Figure S8** Linkage disequilibrium (LD) evolution in the EtNAM subset.

**Figure S9** Phenotypic distribution of days to booting (DB), heading (DH), maturity (DM), and plant height (PH) in the EtNAM population subset.

**Table S1** Founders of the EtNAM population.

**Table S2** Genotyping statistics for the EtNAM subset.

**Table S3** Physical position of markers on wild emmer genome.

**Table S4** Distortion in founder contributions to EtNAM RIL families.

**Table S5** Outcome of the GWA study conducted on phenology traits and plant height.

**Table S6** Gene models within ± 500 Kb of QTNs.
