# Supplementary figures and images for: A large nested association mapping population for breeding and quantitative trait locus mapping in Ethiopian durum wheat
Source: Plant Biotechnol J. 2019 Feb 9;17(7):1380–93. doi: 10.1111/pbi.13062 (PMC6576139; doi:10.1111/pbi.13062)

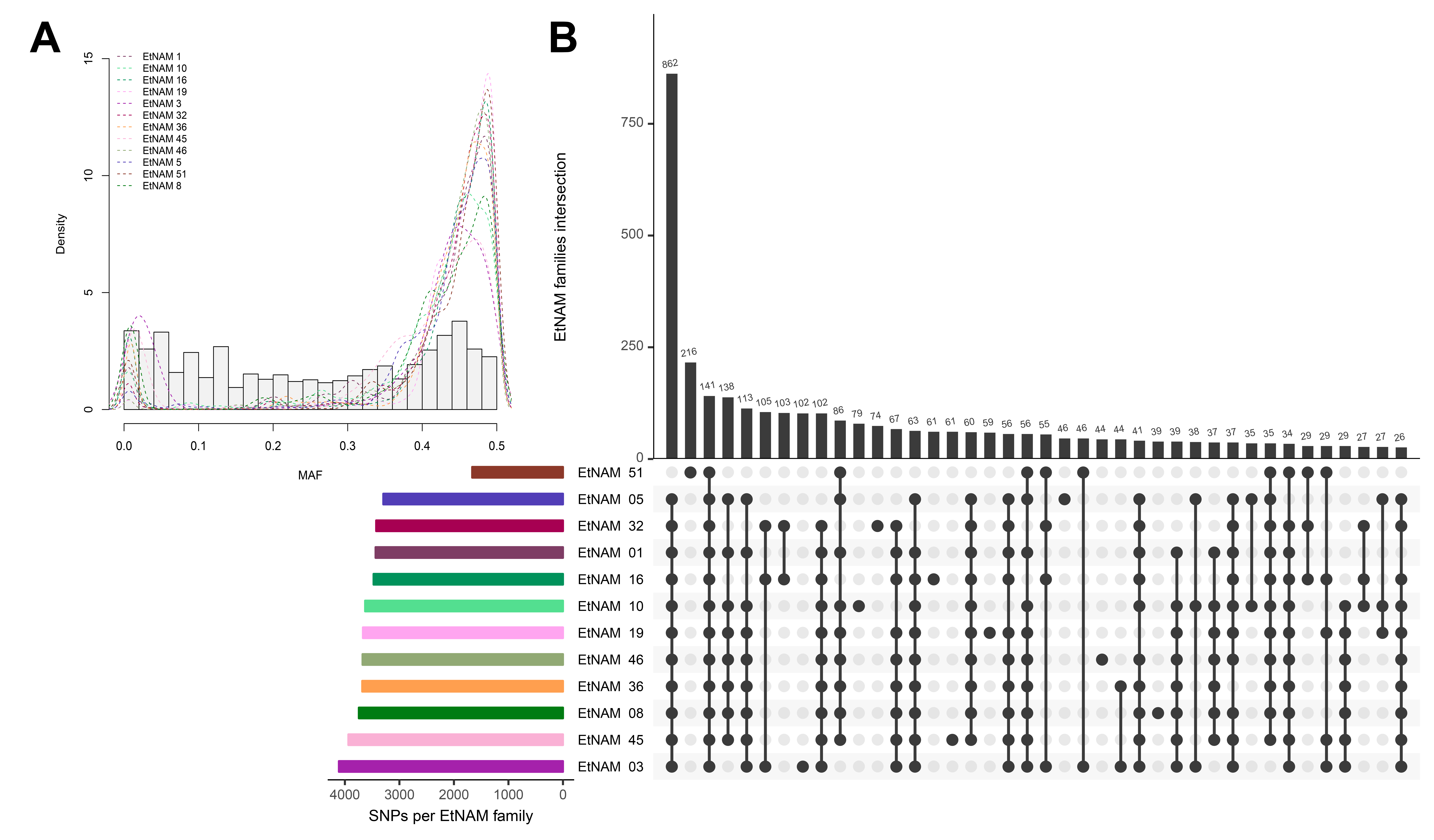

Supplement: Supplementary file 1 — Figure S1 Features of the marker data produced in the EtNAM subset. [file PBI-17-1380-s015.tif]

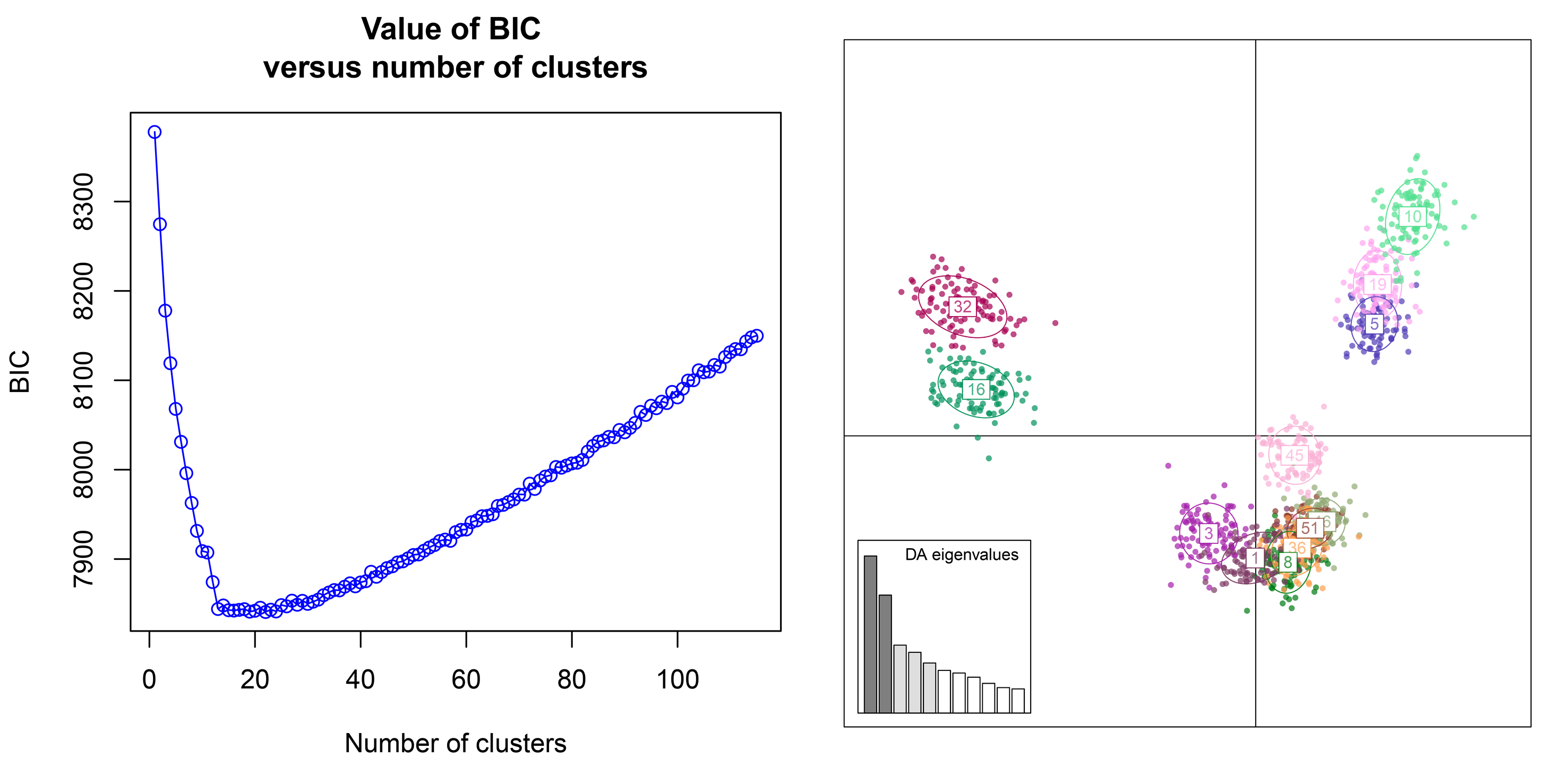

Supplement: Supplementary file 4 — Figure S4 Structure in the EtNAM subset. [file PBI-17-1380-s002.tif]

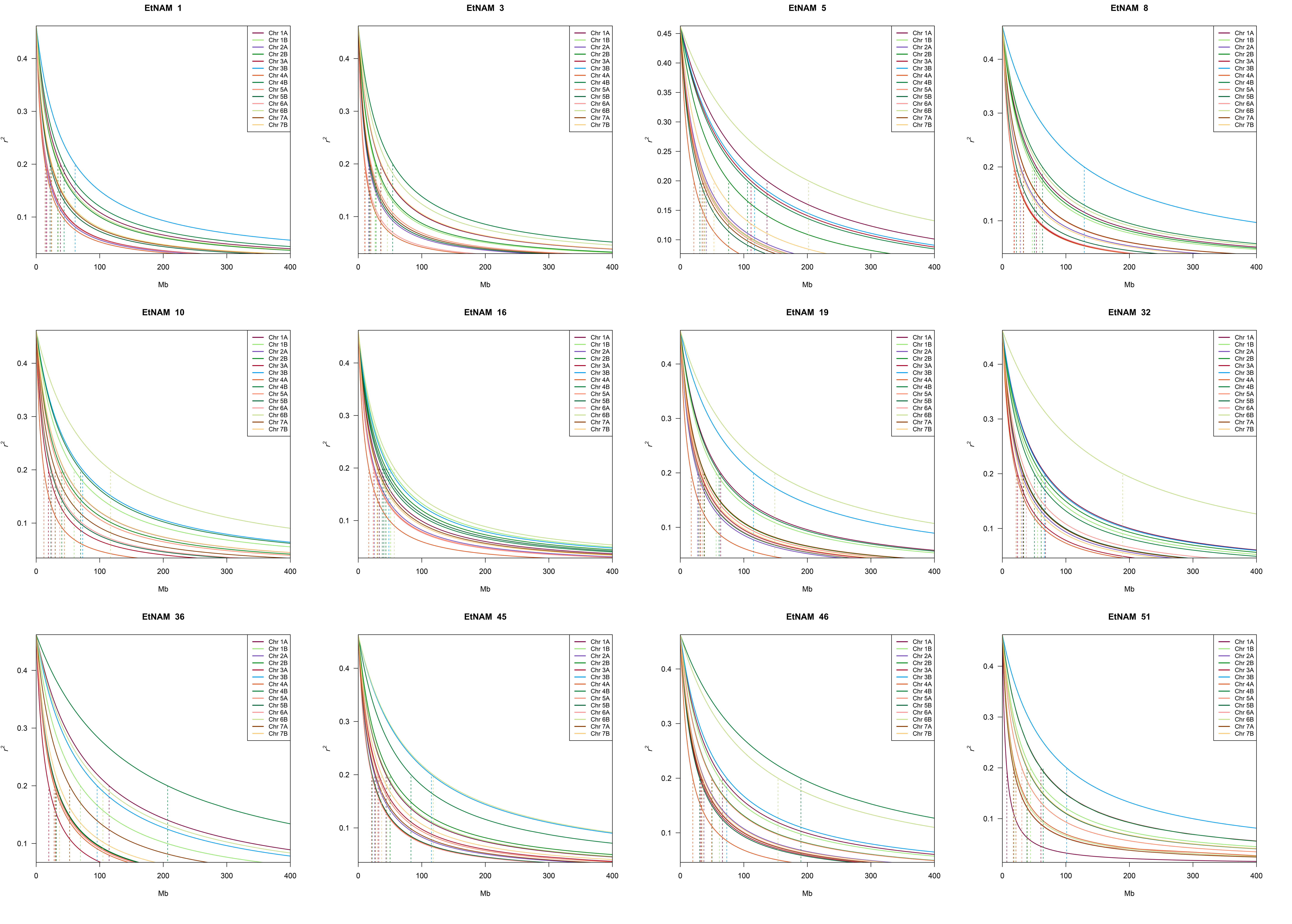

Supplement: Supplementary file 6 — Figure S6 Chromosome‐specific linkage disequilibrium (LD) decay as a function of physical distance in each of the EtNAM families. [file PBI-17-1380-s016.tif]

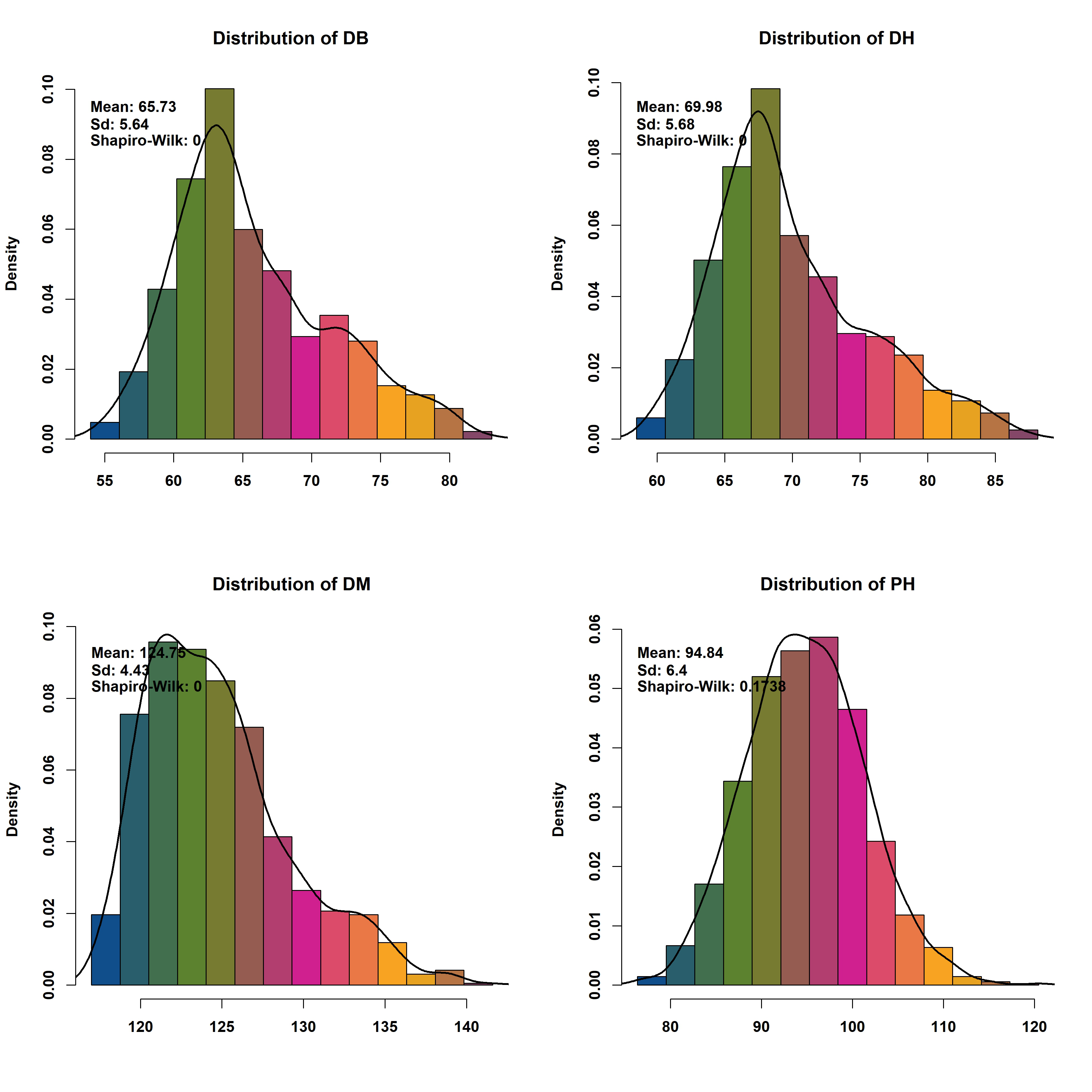

Supplement: Supplementary file 9 — Figure S9 Phenotypic distribution of days to booting (DB), heading (DH), maturity (DM), and plant height (PH) in the EtNAM population subset. [file PBI-17-1380-s005.tif]
