# Supplementary material for: A large nested association mapping population for breeding and quantitative trait locus mapping in Ethiopian durum wheat
Source: Plant Biotechnol J. 2019 Feb 9;17(7):1380–93. doi: 10.1111/pbi.13062 (PMC6576139; doi:10.1111/pbi.13062)
Supplement: Supplementary file 16 — Supplementary Caption [file PBI-17-1380-s012.docx]

**Supplementary Materials**

**Fig. S1** Features of the marker data produced in the EtNAM subset. (A) Minor allele frequency (MAF) distribution in the EtNAM. The family-specific MAF is represented by density lines colored according to the legend. The MAF distribution in the EtNAM subset is represented by the histogram bars in the background. (B) Overlap of SNP sets across EtNAM families. In the lower panel, EtNAM families are sorted by the number of polymorphic markers, and sharing patterns of SNPs are represented by connected dark points. In the top panel, the number of SNPs shared by each group of EtNAM families, as per the lower panel, is reported in decreasing sizes of SNP sets.

**Fig S2** Principal component analysis of molecular diversity in the EtNAM subset. In the left panel, the combination of PC 1 to 4, showing RIL clustering. In the right panel, percental amount of variance (y-axis) explained by each subsequent PC (x-axis).

**Fig S3** Distribution of markers and SNPs in the EtNAM subset. A barplot is produced for each chromosome (Chr), showing as gray bars the number of markers (y axis) in ordered genomic bins (x axis). Red bars are overlaid on grey bars showing the number of markers polymorphic in each genomic bin.

**Fig S4** Structure in the EtNAM subset. The panel on the left represents the evolution of the Bayesian information criteria (BIC) with increasing number of genetic clusters postulated (maximum explanatory value at the lower BIC). The panel on the right represents the outcome of a discriminant analysis of principal components (DAPC). RILs grouping according to the first two DA is reported, with colors according to Fig. 3 and numbers according to EtNAM family naming.

**Fig S5** Marker loadings on the first three DAs deriving from the DAPC. Markers are represented as points colored by chromosome as per legend. On the x-axis, the genomic position in Mbp. On the y-axis, the loading, *i.e.* contribution of each marker to the DA.

**Fig S6** Choromosome-specific linkage disequilibrium (LD) decay as a function of physical distance in each of the EtNAM families. Each plot represents LD decay in one of the families, as *r^2^* (y axis) over physical distance in Mbp (x axis), with colors according to legend. The distance of null LD (*r^2^* = 0.2) is represented by vertical dashed lines for each Chr.

**Fig S7** Pairwise linkage disequilibrium (LD) in the EtNAM subset. Markers are ordered according to their physical positions. Black segments project to their map position (whose length is reported). Increasing values of *r^2^* are reported in increasing shades of red according to legend for each Chr.

**Fig S8** Linkage disequilibrium (LD) evolution in the EtNAM subset. For each Chr, LD measures are averaged in genomic bins and represented as a continuous line with Chr-specific color. On the x-axis, the Mb positions across the Chr. Molecular markers available on each Chr are represented by gray ticks at the bottom of the plot, in the corresponding physical positions.

**Fig S9** Phenotypic distribution of days to booting (DB), heading (DH), maturity (DM), and plant height (PH) in the EtNAM population subset. For each distribution mean, standard deviation, and the significance of a Shapiro-Wilkinson test for normality are given.

**Table S1** Founders of the EtNAM population. The table reports the accession name for each of the genotypes used in the EtNAM crosses (accession names as per EBI sample codes). The species, Region and Ethiopian zone of origin for each founder are also reported. When present, the phenotypic attributes contributing to the choice of founder genotypes are reported.

**Table S2** Genotyping statistics for the EtNAM subset. For each family in the subset, the table reports the number of polymorphic SNPs (SNPs), the proportion of heterozygous markers (Het), and the proportion of markers with failed allele calls (Failed; “N” in the genotyping matrix).

**Table S3** Physical position of markers on wild emmer genome. For each marker, chromosome (Chr) and position in bp (Pos) are reported. Chromosomes are listed in a numerical code from 1 to 15, where Chr 1, 2, 3, …, 14 correspond to Chr 1A, 1B, 2A, …, 7B, and Chr15 corresponds to Chr Unknown of the wild emmer genome assembly

**Table S4** Distortion in founder contributions to EtNAM RIL families. For each considered marker, the table reports the chromosome (Chr), position in Mb (Mb), and the associated p-value for a chi squared test for goodness of fit in each of the EtNAM families. The expected contribution of the two parental alleles is 50% at each locus. When a specific marker is not polymorphic or not typed in one of the populations, a missing value (NA) is reported. Chromosomes are listed in a numerical code from 1 to 14, where Chr 1, 2, 3, …, 14 correspond to Chr 1A, 1B, 2A, …, 7B.

**Table S5** outcome of the GWA study conducted on phenology traits and plant height. For each marker the name (marker), the position according to the *T. dicoccoides* genome assembly is given. Traits are then reported one column after the other, with the effect estimated by the model and the significance of the association (p value). Trait names as per main text. Chromosomes are listed in a numerical code from 1 to 14, where Chr 1, 2, 3, …, 14 correspond to Chr 1A, 1B, 2A, …, 7B.

**Table S6** Gene models within ±500 Kb of QTNs. For each gene, the table reports the trait, the Chr, the coordinates, and the identifier in the wild emmer annotation v1.0.59
